# The Phœnician and Carthaginian Influence

**Published:** 1896-09-26

**Authors:** 


### Modern Sociology.

### THE PHŒNICIAN AND CARTHAGINIAN INFLUENCE.

Carthage has been compared to a falling star among the nations. As the Republic of the United States of America has been called the eldest daughter of England, so Carthage might be called the eldest daughter of Tyre, the daughter herself of Sidon, the oldest of the original great Phœnician cities on the seaboard of Canaan. Although for seven hundred years or more the greatest maritime and commercial state of the ancient world, hardly anything is known of the art, science, or literature of this great people except through alien sources, and lately by modern excavations. The only specimen of the language of Carthage, even, is preserved in a play written by a citizen of her greatest enemy, and eventually her destroyer, the Roman Terence. And yet this nation and its Phœnician ancestors exercised a powerful and enduring influence upon the destinies of mankind. As we remarked in a former article, Phœnicia will never be forgotten, because the world received its alphabet from her. Printing has by some been considered the greatest of modern inventions, but what would printing be without letters? We shall endeavour to show briefly how this State, the very embodiment of Commerce in the ancient world, influenced by its example and destiny the growth of those social ideas, which seem to be coming to maturity, only in our own times. Readers of the Bible can easily understand the position of the Phœnicians in the ancient world by turning to the chapters in Ezekiel xxvii. and xxviii., which describes the commerce and wealth of Tyre and Sidon in those days, centuries before the Christian era. We will only quote one or two verses, as the Bible is the most easily accessible of all books to those who wish to study it—"And say unto Tyrus, O thou that art situate at the entry of the sea, which art a merchant of the people for many isles"; and in the next chapter, "With thy wisdom and with thy understanding, thou hast gotten thee riches, and hast gotten gold and silver into thy treasures; by thy great wisdom and by thy great traffick hast thou increased thy riches, and thine heart is lifted up because of thy riches." The whole description given in these two chapters is a marvellously vivid one, and might well be applied to one or two famous modern nations. Like the Hebrews, the Phœnicians were, it may be said, the first of the Eastern nations who faced the west looking over the Mediterranean or Great Sea, as it was then called, just as Liverpool now looks over the Atlantic towards America. They called their own land "Canaan" or "the plain," but were called Phœnicians by the Greeks "the land of purple," or "the land of the red men." The field of their commerce extended from Western Africa and Cornwall on the West, to the Eastern Coast of India; and they, in fact, did all the carrying trade of the ancient world. The influence of such a nation is peculiarly interesting, especially to Englishmen, who in some respects hold a similar position in the modern world. Indeed, the first Napoleon in one of his speeches described England as the modern Carthage, against whom he was struggling.

The Carthaginians more than any other people of Oriental origin approached in the similarity of their political institutions to the nations of the West, a fact noticed both by Aristotle and Polybius as well as by other ancient writers. Like Rome and the Greek cities they possessed to a certain extent representative municipal government. Although chiefly merchants and traders, they took a keen interest in agriculture, and works written on that subject in their language were translated into Latin for the benefit of the people of Italy by command of the Roman Senate. They represented the materialistic side of the Semitic character, as the Hebrews in their highest development represented the spiritual, although in the latter race also there has been a constant struggle between these two elements from the days when the golden calf was made by Aaron to the present age. On the one side have been the goodly fellowship of the Prophets, the glorious company of the Apostles, and the noble army of Martyrs; on the other, high financiers, and all the classes who generally take the more material view of life. The chief interest to the student of sociology in the history of the Phœnicians and the Carthaginians lies in the fact that they of all the great nations in the ancient world were the most exclusively devoted to commerce and all that concerns commerce. They came into hostile collision with the Greeks on many occasions, but ultimately each nation recognised the other's sphere of influence, to use a familiar modern political expression. Later on in their history they fought a long and deadly duel with Rome, which eventually ended in their annihilation as an independent nation. Yet, as the greatest commercial and colonising people of their own period, their political and social history must always be interesting as that of a State which for between six and seven hundred years held a foremost place amongst the nations of the world. In a later article we hope to be able to show what influence the third of the great Semitic nations—the Arabic—with its own, as it were, special religion, Mohammedanism has had upon the development of social ideas. The Arab may be said to stand between the Hebrew and the Phœnician, having much of the spiritual character of the former combined with a good deal of the commercial instinct of the latter. The world will always be grateful to the Phœnicians for the spread of the knowledge of letters, and probably many other things which we cannot now trace which these daring and enterprising mariners of the old world spread abroad in their venturesome journeys from sea to sea. Perhaps the most peculiarly interesting fact in their history to the modern student is their treatment of the subject races, with whom they did not combine, and whom they could not assimilate. Another point of great interest is the fact that, as far as we know, there were none of the internecine conflicts between the various Phœnician cities, which looked up to Carthage as their head, like those which did so much to ruin the prosperity of the Greek cities, and which ultimately led to the loss of freedom in Greece. There must have been some elements of great power and wisdom in a State which could bring about a permanent peace between so many conflicting interests.

